# The prevalence of impulsive compulsive behaviors in patients treated with apomorphine infusion: a retrospective analysis

**DOI:** 10.1590/0004-282X-ANP-2020-0522

**Published:** 2021-11-30

**Authors:** Pedro Barbosa, Atbin Djamshidian, Andrew John Lees, Thomas Treharne Warner

**Affiliations:** 1 University College London, Institute of Neurology, Reta Lila Weston Institute of Neurological Studies, London, UK. University College London Institute of Neurology Reta Lila Weston Institute of Neurological Studies London UK; 2 University College London, Institute of Neurology, Queen Square Brain Bank for Neurological Disorders, London, UK. University College London Institute of Neurology Queen Square Brain Bank for Neurological Disorders London UK; 3 Innsbruck Medical University, Department of Neurology, Innsbruck, Austria. Innsbruck Medical University Department of Neurology Innsbruck Austria

**Keywords:** Parkinson Disease, Compulsive Behavior, Impulsive Behavior, Disruptive, Impulse Control, and Conduct Disorders, Apomorphine, Doença de Parkinson, Comportamento Compulsivo, Comportamento Impulsivo, Transtornos Disruptivos, de Controle do Impulso e da Conduta, Apomorfina

## Abstract

**Background:**

Impulsive compulsive behaviors (ICBs) can affect a significant number of Parkinson’s disease (PD) patients.

**Objective:**

We have studied brain samples from a brain bank of PD patients who received apomorphine via continuous infusion in life to assess the prevalence and outcome of ICBs.

**Methods:**

A search on the Queen Square Brain Bank (QSBB) database for cases donated from 2005 to 2016 with a pathological diagnosis of idiopathic PD was conducted. Notes of all donors who used apomorphine via continuous infusion for at least three months were reviewed. Clinical and demographic data were collected, as well as detailed information on treatment, prevalence and outcomes of ICBs.

**Results:**

193 PD cases, 124 males and 69 females, with an average age at disease onset of 60.2 years and average disease duration of 17.2 years were reviewed. Dementia occurred in nearly half of the sample, depression in one quarter, and dyskinesias in a little over 40%. The prevalence of ICBs was 14.5%. Twenty-four individuals used apomorphine infusion for more than three months. Patients on apomorphine had younger age at disease onset, longer disease duration, and higher prevalence of dyskinesias. The prevalence of *de novo* ICB cases among patients on apomorphine was 8.3%. Apomorphine infusion was used for an average of 63.1 months on an average maximum dose of 79.5 mg per day. Ten patients remained on apomorphine until death.

**Conclusions:**

Apomorphine can be used as an alternative treatment for patients with previous ICBs as it has low risk of triggering recurrence of ICBs.

## RESUMO

**Antecedentes:**

Comportamentos impulsivo-compulsivos (CICs) podem acometer uma parcela significativa de indivíduos com doença de Parkinson (DP).

**Objetivo:**

Nós estudamos amostras de tecido cerebral de uma população de pacientes com DP de um banco de cérebros que receberam apomorfina por infusão contínua em vida, com a finalidade de avaliar a prevalência e o desfecho dos CICs. Métodos: Uma pesquisa no banco de dados do Banco de Cérebros de Queen Square foi conduzida à procura de doações recebidas entre 2005 e 2016 com diagnóstico anatomopatológico de DP idiopática. Os prontuários de todos os doadores que usaram apomorfina por infusão contínua por um período mínimo de três meses foram revisados. Dados clínicos e demográficos foram coletados, assim como informações detalhadas sobre o tratamento, prevalência e desfecho dos CICs.

**Resultados:**

193 casos de DP, 124 do sexo masculino e 69 do sexo feminino, com idade média de início da doença de 60,2 anos e tempo médio de duração da doença de 17,2 anos, foram revisados. Aproximadamente metade dos casos apresentaram demência, um quarto depressão, e um pouco mais de 40% discinesias. A prevalência de CICs foi 14,5%. Vinte e quatro indivíduos usaram infusão de apomorfina por mais de três meses. Os pacientes que usaram apomorfina apresentaram DP mais cedo, maior duração da doença, e uma maior prevalência de discinesias. A prevalência de novos casos de CICs entre pacientes usando apomorfina foi de 8,3%. Infusão de apomorfina foi usada em média por 63,1 meses a um dose máxima média de 79,5 mg por dia. Dez pacientes permaneceram usando apomorfina até o óbito.

**Conclusões:**

Apomorfina pode ser usada como opção de tratamento alternativo para pacientes que apresentarem CICs no passado considerando seu baixo risco de causar recorrência de CICs.

## INTRODUCTION

Impulsive compulsive behaviors (ICBs), such as dopamine dysregulation syndrome (DDS), hypersexuality, pathological gambling, compulsive shopping, compulsive eating and punding are relatively common behavioral complications that can affect from 14 to 36% of patients with Parkinson’s disease (PD)^1^^,^^2^. The main risk factors for the development of ICBs are male sex, young age at PD onset, and dopaminergic treatment. Even though levodopa has also been associated with these abnormal behaviors, the main risk factor is the use of dopamine agonists^1^. In a population of PD patients receiving one dopamine agonist (DA) for at least six months, the prevalence of ICBs reached 39%^3^.

Neuroimaging studies have shown that excessive dopaminergic release in the ventral striatum occurs in individuals with DDS^4^ and other ICBs^5^. However, data from clinical studies suggest that excessive stimulation of dopaminergic D3 receptors, abundantly expressed in the nucleus accumbens^6^, might also play a role^7^.

Apomorphine is a dopamine agonist with preferential binding to D1 and D2 dopaminergic receptors^8^. Therefore, studies assessing the development of ICBs in PD patients on apomorphine via continuous infusion could shed some light on the pathophysiology of ICBs. Initial results suggest a lower proclivity of apomorphine to trigger these abnormal behaviors, indicating that either pulsatile rather than continuous stimulation of dopaminergic receptors is associated with ICBs, or that stimulation of D3 receptors is a key factor, or perhaps a combination of both^9^^-^^11^.

We conducted a retrospective analysis to assess the prevalence and outcome of ICBs in brain samples of a population of PD patients treated with apomorphine continuous infusion who donated their brains to the Queen Square Brain Bank (QSBB), London, UK.

## METHODS

We searched the QSBB database for consecutive cases donated from 2005 to 2016 with a pathological diagnosis of idiopathic Parkinson’s disease. Subsequently, all donors who had received treatment with apomorphine via continuous infusion for at least 3 months were identified and case files separated for a detailed review of notes. All files were reviewed by a neurologist with expertise in movement disorders (PB). Clinical and demographic data were collected with emphasis on dopaminergic treatment and neuropsychiatric complications, as well as indication for apomorphine, dose changes, pre-existing ICBs and outcome after apomorphine, and new-onset ICBs. 

The diagnosis of impulse control disorders was based on the Diagnostic and Statistical Manual of Mental Disorders (DSM-IV) and the diagnosis of DDS was based on previously published diagnostic criteria^1^.

All variables were tested for normality and statistical tests were chosen accordingly. Parametric data were compared using the unpaired t-test and non-parametric data, the Mann-Whitney U test. Proportions were analyzed using the chi-square test. Data was analyzed using SPSS 22^(^.

## RESULTS

The database search returned 193 cases with a pathological diagnosis of idiopathic PD from 2005 to 2016, 124 males and 69 females. Clinical and demographic data are summarized in Table 1.

**Table 1.** Clinical and demographic characteristics of the entire cohort.

	N = 193
Females (%)	69 (35.8%)
Age at PD onset (years)	60.2 ((10.9; 28 -88)
Disease duration (years)	17.2 ((8; 3 -39)
Age at death (years)	77.5 ((7.7; 52 -96)
Dementia (%)	87 (45%)
Depression (%)	47 (24.4%)
Dyskinesias (%)	80 (42%)
ICBs (%)	28 (14.5%)
Apomorphine infusion (%)	24 (12.4%)

PD: Parkinson’s disease; ICBs: impulsive compulsive behaviors.

Twenty-eight patients were diagnosed with ICBs: 22 with one ICB (12 with DDS, 9 with hypersexuality, 1 with pathological gambling, and 1 with compulsive shopping) and 6 with multiple ICBs (3 with DDS and hypersexuality, 1 with DDS and pathological gambling, and 1 with hypersexuality and punding).

Twenty-four patients used apomorphine infusion for more than three months. Another 5 patients underwent an apomorphine trial: 2 could not tolerate the drug because of side effects, 2 were unable to operate the pump, and one had a negative apomorphine challenge.

For statistical analysis, patients were divided into two groups based on the use of apomorphine infusion for more than three months (APO+ and APO-). Sex distribution was similar in both groups. Compared to the patients who did not use apomorphine, the patients on the APO+ group had younger age at disease onset, longer disease duration, and died at an earlier age. There was no difference in the prevalence of dementia and depression between groups, but dyskinesias were significantly more prevalent in the APO+ group (Table 2).

**Table 2.** Comparison between groups that used or not apomorphine infusion for more than 3 months.

	APO+ (N = 24)	APO-(N = 169)	p
Females (%)	9 (37.5%)	60 (35.5%)	0.824*
Age at PD onset (years)	51.33 ((8)	61.56 ((10.7)	<0.001**
Disease duration (years)	22.88 ((6.25)	16.49 ((7.9)	<0.001***
Age at death (years)	74.2 ((7.5)	78 ((7.6)	0.016***
Dementia (%)	11 (45.8%)	76 (44.9%)	1.000*
Depression (%)	9 (37.5%)	38 (22.4%)	0.092*
Dyskinesias (%)	23 (95.8%)	58 (34.3%)	<0.001*

PD: Parkinson’s disease; *Chi-square test; **unpaired t-test; ***Mann-Whitney test; Significant results are in bold.

All patients who used apomorphine infusion for more than three months had their full set of notes reviewed. Apomorphine pump was used for an average of 63.1 months. The maximum dose of apomorphine reached was on average 77.9 mg per day. Apomorphine was discontinued prematurely in 14 cases: in 2 because of inadequate control of PD symptoms, in 1 because of excessive dyskinesias, in 8 because of side effects, in 1 because of technical issues, and in 2 because of lack of benefit. Ten patients remained on apomorphine until death (Table 3).

**Table 3.** Data on apomorphine use by Parkinson’s disease patients.

	N = 24
Apomorphine treatment duration	63.1 months ((54.2; 3 -216)
Apomorphine maximum daily dose	77.9 mg ((36.9; 15 -150)
Apomorphine discontinued	14 (58.3%)
Use of intermittent injections of apomorphine	Total -13 (54.1%) Concomitant -3 (12.5%)
Use of dopamine agonists	Total -21 (87.5%) Concomitant -11 (45.8%)

Three patients in the APO+ group received surgical treatment for PD, 2 pallidotomy and 1 deep brain stimulation (DBS), initially of the subthalamic nucleus and 2 years later of the globus pallidus internus. One patient received an experimental treatment with fetal mesencephalic transplant. In the APO+ group, thirteen patients also received treatment with intermittent injections of apomorphine. Most used the pen only before being prescribed the pump but 3 patients continued with the pen after being prescribed apomorphine via continuous infusion (Table 3).

Only 3 patients in the APO+ group did not use oral/transdermal dopamine agonists. Of the 21 patients that used dopamine agonists, 11 used it concomitantly with apomorphine infusion. The most common agonist used in the APO+ group was pergolide (used by 10 patients), followed by ropinirole (8 patients), rotigotine (7 patients), cabergoline (7 patients), pramipexole (2 patients), and lysuride (1 patient). DA dose was calculated in levodopa equivalent daily dose (LEDD) as previously described^12^. Regarding pathological diagnosis, 8 patients were classified as Braak stage 5 and 16 as Braak stage 6 (Table 3)^13^. 

**Figure 1.**
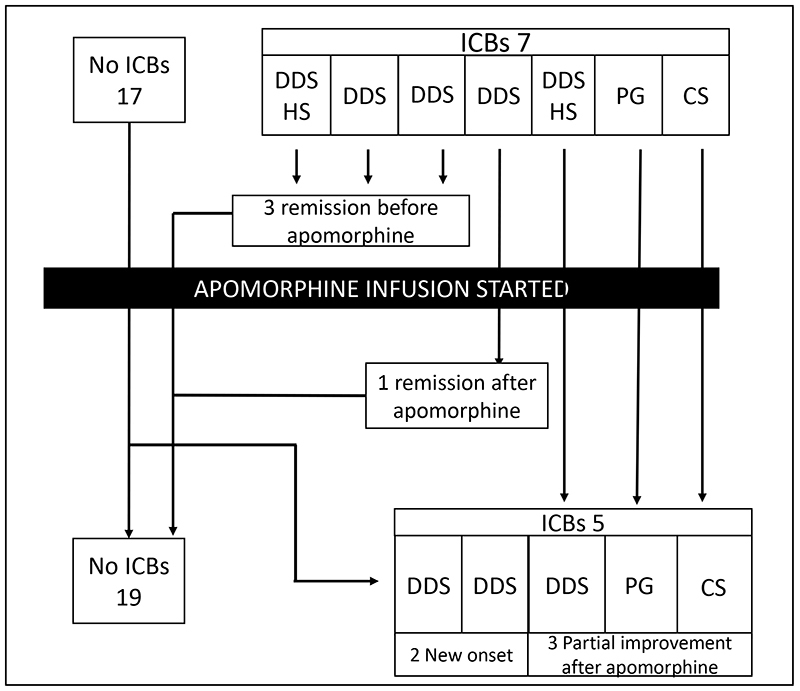
Patients on apomorphine infusion and development of ICBs. Seven patients had ICBs before apomorphine. Three improved completely and 3 improved partially before starting this medication, and none of these patients worsened after the pump was prescribed. One patient with DDS improved completely after apomorphine treatment. Two patients had new onset DDS during apomorphine treatment.

ICBs: impulsive compulsive behaviors; DDS: dopamine dysregulation syndrome; HS: hypersexuality; PG: pathological gambling; CS: compulsive shopping.

Although a larger number of individuals in the APO+ group developed ICBs, there were only 2 *de novo* cases of ICBs during apomorphine use. Seven patients developed behavioral addictions before starting treatment with the apomorphine pump: two of them had DDS and hypersexuality, one improved completely before starting the pump and another improved partially, remaining with mild DDS after apomorphine; two other patients with DDS improved completely before the pump and did not experience recurrence on apomorphine; one patient with DDS improved completely after starting apomorphine infusion; one patient with pathological gambling improved partially before apomorphine and did not worsen with the pump; and one patient with compulsive shopping improved partially after treatment with apomorphine infusion (Figure 1).

Two patients developed new onset DDS after being prescribed the pump. In one case, the abnormal behavior started during the first six months of continuous apomorphine infusion and led to a confusional state after a large dose increase by the patient. DDS improved completely after adjustment of the medications and did not recur during another five years of pump use. The other new onset DDS case was a patient that started to overdose on levodopa approximately 6 years after the pump was prescribed. At that time, he was taking up to 48 tablets of levodopa/carbidopa 50/12.5 mg per day. This was the only patient with ICBS that had never been exposed to an oral or transcutaneous DA and remained symptomatic until death (Figure 1).

One patient developed DDS nine years after discontinuing treatment with apomorphine infusion while receiving treatment with levodopa and cabergoline. Details on the patients who developed ICBs are shown in Figure 1.

## DISCUSSION

A retrospective analysis of the prevalence and outcomes of ICBs among PD patients from the QSBB that used apomorphine via continuous infusion for more than three months was conducted. 

PD affects men more than women, but the male to female ratio of 1.8:1 was slightly higher than that published in the literature^14^. Although the average age at PD onset of the entire cohort was compatible with the literature, patients that used apomorphine infusion developed PD much earlier, at approximately 50 years of age. This is similar to other cohorts of PD patients on apomorphine^15^^,^^16^ and probably reflects the fact that younger patients are more likely to develop motor fluctuations earlier, one of the main indications for apomorphine continuous infusion^17^. Disease duration of the APO+ group was six years longer than the average of the entire cohort. The longer disease duration probably reflects the predominance of younger patients in the APO+ group as these patients may have slower disease progression^18^.

Life expectancy in the UK in 2014 was 79.5 years for males and 83.2 years for females (https://www.ons.gov.uk/), while the average age at death in this cohort was 77.5 years, slightly lower than the national average. This is compatible with previous published research showing that PD patients have increased mortality rates after 10 years of disease progression compared to the general population^19^. Age at death was lower in the APO+ group but this was partly compensated by the longer disease duration in this group. 

Dementia developed in 45% of patients, a similar prevalence to that found in the CamPAIGN study^20^ but slightly higher than a meta-analysis published in 2005^21^. Depression was present in approximately 24% of individuals, in line with previously published data^22^. The prevalence of both dementia and depression was not influenced by the use of apomorphine. 

Levodopa-induced dyskinesias affected 42% of the patients, less than what is expected for a population with more than 17 years of disease progression^23^. It is possible that this finding is an underestimate associated with retrospective data collection. Of the APO+ group, all patients but one developed dyskinesias, a much higher prevalence than what is expected for PD patients. Data from the literature shows that younger age at PD onset, longer disease duration, and longer exposure to levodopa are risk factors for the development of dyskinesias and could explain this finding^23^. A recently published study found that patients with PD and dyskinesias have a higher prevalence of ICBs than the general PD population, suggesting the presence of shared mechanisms between both phenomena^24^.

The mean maximum daily dose of apomorphine in this study was 77.9 mg per day, higher than the dose reported by another study we conducted with living patients^25^ but still lower than the 98 mg reported in the early 2000s in our centre^15^. The pump was used for an average of 5.2 years and was well tolerated by the majority of patients. However, a little over half of the patients had to discontinue apomorphine, a third of them because of side effects. The fact that nearly 40% of the patients remained on the pump until death shows that for some patients apomorphine remains a reliable treatment option until final stages of the disease.

The prevalence of ICBs found was lower than that published in the literature^2^. Even though only donations received after the year 2005 when clinical awareness of ICBs was more widespread were included, it is possible that some ICB cases in our cohort were not detected, as patients with ICBs are less likely to spontaneously disclose these abnormal behaviours^26^. Another possible explanation for the lower prevalence of ICBs is the relatively high number of patients with cognitive impairment, since a previous study has reported a lower prevalence of impulse control disorders in individuals with Parkinson’s disease dementia^2^.

Data from studies on addiction show that drugs that can cause a rapid increase in dopamine release in the dopaminergic reward pathway have stronger reinforcing properties and are more likely to cause addiction^27^. Whether the different methods of apomorphine delivery, intermittent injections or continuous infusion, are more or less likely to cause DDS or other ICBs is still unclear. Nearly half of the patients in the APO+ group received treatment with apomorphine intermittent injections at some point of their disease course. However, only 3 remained on this medication after starting the pump. The literature lacks data on the propensity of apomorphine delivered as intermittent injections to trigger ICBs and our small sample does not allow us to draw any conclusions.

ICBs have also been associated with excessive stimulation of D3 receptors, but this conclusion has been drawn mainly by findings from clinical studies showing that DAs are the main risk factor for the development of ICBs^1^^,^^3^ and that these drugs have strong affinity for D3 receptors^7^. Apomorphine is a dopamine agonist with different pharmacological profile as it stimulates mainly D1 and D2 receptors, akin to levodopa^8^. A few studies with PD patients on apomorphine infusion have been published and the initial results indicate a lower prevalence of ICBs compared to oral DAs^10^^,^^11^^,^^25^^,^^28^. However, these results need to be confirmed with randomized clinical trials.

Our finding that patients on apomorphine had a lower prevalence of ICBs suggests that apomorphine infusion is not commonly associated with ICBs. The majority of patients that used apomorphine infusion did not develop ICBs. In three patients in whom ICBs had improved before apomorphine infusion, the problem did not recur during treatment, and in 4 patients that were previously symptomatic, the situation improved after apomorphine infusion, completely in 1 and partially in 3. Both the pharmacological profile and delivery by continuous infusion might contribute to apomorphine being less likely to trigger ICBs.

We report 2 new-onset DDS in patients on continuous infusion of apomorphine. Although the prevalence of ICBs was similar to what has been found by other authors studying infusion therapies in PD^11^^,^^28^, it is possible that other dopaminergic medication contributed to the development of DDS, as levodopa use appears to be the most important risk factor for the development of DDS^29^. The fact that complete improvement occurred in one of the cases despite remaining on apomorphine infusion, and that ICBs occurred in another case 6 years after the pump was prescribed, supports this hypothesis. One limitation of this paper was the inclusion of DDS and other types of ICBs under the same group. While there are pathophysiological features common to these conditions^3^, DDS and other types of ICBs have different risk factors. 

The main advantage of using a brain bank cohort is the ability to confirm the diagnosis of PD through *postmortem* examination. It is known that even in specialized centers, a small proportion of patients can be misdiagnosed with PD^30^. The main disadvantage is that clinical information is acquired retrospectively, and the quality of data is heavily dependent on the thoroughness of hospital records. Considering that all patients in this cohort were seen by consultant neurologists regularly, we believe that the quality of the data was appropriate for the purposes of this study. Another potential issue is the small sample of patients using apomorphine infusion. Even though our data suggests that apomorphine is not usually associated with ICBs, larger studies are needed to confirm these findings.

In conclusion, continuous infusion of apomorphine can be used as an alternative treatment option for patients with advanced PD who previously developed ICBs, as it has a low risk of triggering recurrence of ICBs.

## References

[B1] Weintraub D, Koester J, Potenza MC, Siderowf AD, Stacy M, Voon V (2010). Impulse control disorders in Parkinson disease: a cross-sectional study of 3090 patients. Arch Neurol.

[B2] Biundo R, Weis L, Abbruzzese G, Calandra-Buonaura G, Cortelli P, Jori MC (2017). Impulse control disorders in advanced Parkinson's disease with dyskinesia: the ALTHEA study. Mov Disord.

[B3] Garcia-Ruiz PJ, Castrillo JCM, Alonso-Canovas A, Barcenas AH, Vela L, Alonso PS (2014). Impulse control disorder in patients with Parkinson's disease under dopamine agonist therapy: a multicentre study. J Neurol Neurosurg Psychiatry.

[B4] Evans AH, Pavese N, Lawrence AD, Tai YF, Appel S, Doder M (2006). Compulsive drug use linked to sensitized ventral striatal dopamine transmission. Ann Neurol.

[B5] O'Sullivan SS, Wu K, Politis M, Lawrence AD, Evans AH, Bose SK (2011). Cue-induced striatal dopamine release in Parkinson's disease-associated impulsive-compulsive behaviours. Brain.

[B6] Gurevich EV, Joyce JN (1999). Distribution of dopamine D3 receptor expressing neurons in the human forebrain: comparison with D2 receptor expressing neurons. Neuropsychopharmacology.

[B7] Seeman P (2015). Parkinson's disease treatment may cause impulse-control disorder via dopamine D3 receptors. Synapse.

[B8] Fahn S, Jankovic J, Hallett M (2011). Principles and practice of movement disorders.

[B9] Todorova A, Martinez-Martin P, Martin A, Rizos A, Reddy P, Chaudhuri KR (2013). Daytime apomorphine infusion combined with transdermal Rotigotine patch therapy is tolerated at 2 years: A 24-h treatment option in Parkinson's disease. Basal Ganglia.

[B10] Magennis B, Cashell A, O'Brien D, Lynch T (2012). An audit of apomorphine in the management of complex idiopathic Parkinson's disease in Ireland. Movement Disord.

[B11] Martinez-Martin P, Reddy P, Katzenschlager R, Antonini A, Todorova A, Odin P (2015). EuroInf: a multicenter comparative observational study of apomorphine and levodopa infusion in Parkinson's disease. Mov Disord.

[B12] Tomlinson CL, Stowe R, Patel S, Rick C, Gray R, Clarke CE (2010). Systematic review of levodopa dose equivalency reporting in Parkinson's disease. Mov Disord.

[B13] Braak H, Tredici KD, Rüb U, de Vos RAI, Steur ENHJ, Braak E (2003). Staging of brain pathology related to sporadic Parkinson's disease. Neurobiol Aging.

[B14] Picillo M, Nicoletti A, Fetoni V, Garavaglia B, Barone P, Pellecchia MT (2017). The relevance of gender in Parkinson's disease: a review. J Neurol.

[B15] Manson AJ, Turner K, Lees AJ (2002). Apomorphine monotherapy in the treatment of refractory motor complications of Parkinson's disease: long-term follow-up study of 64 patients. Mov Disord.

[B16] Tyne HL, Parsons J, Sinnott A, Fox SH, Fletcher NA, Steiger MJ (2004). A 10 year retrospective audit of long-term apomorphine use in Parkinson's disease. J Neurol.

[B17] Schrag A, Quinn N (2000). Dyskinesias and motor fluctuations in Parkinson's disease. A community-based study. Brain.

[B18] Wickremaratachi MM, Ben-Shlomo Y, Morris HR (2009). The effect of onset age on the clinical features of Parkinson's disease. Eur J Neurol.

[B19] Diem-Zangerl A, Seppi K, Wenning GK, Trinka E, Ransmayr G, Oberaigner W (2009). Mortality in Parkinson's disease: a 20-year follow-up study. Mov Disord.

[B20] Williams-Gray CH, Mason SL, Evans JR, Foltynie T, Brayne C, Robbins TW (2013). The CamPaIGN study of Parkinson's disease: 10-year outlook in an incident population-based cohort. J Neurol Neurosurg Psychiatry.

[B21] Aarsland D, Zaccai J, Brayne C (2005). A systematic review of prevalence studies of dementia in Parkinson's disease. Mov Disord.

[B22] Reijnders JSAM, Ehrt U, Weber WEJ, Aarsland D, Leentjens AFG (2008). A systematic review of prevalence studies of depression in Parkinson's disease. Mov Disord.

[B23] Zesiewicz TA, Sullivan KL, Hauser RA (2007). Levodopa-induced dyskinesia in Parkinson's disease: epidemiology, etiology, and treatment. Curr Neurol Neurosci Rep.

[B24] Biundo R, Weis L, Abbruzzese G, Calandra-Buonaura G, Cortelli P, Jori MC (2017). Impulse control disorders in advanced Parkinson's disease with dyskinesia: the ALTHEA study. Mov Disord.

[B25] Barbosa P, Lees AJ, Magee C, Djamshidian A, Warner TT (2016). A Retrospective Evaluation of the Frequency of Impulsive Compulsive Behaviors in Parkinson's Disease Patients Treated with Continuous Waking Day Apomorphine Pumps. Mov Disord Clin Pract.

[B26] Perez-Lloret S, Rey MV, Fabre N, Ory F, Spampinato U, Montastruc J-L (2012). Do Parkinson's disease patients disclose their adverse events spontaneously?. Eur J Clin Pharmacol.

[B27] Koob GF, Volkow ND (2010). Neurocircuitry of addiction. Neuropsychopharmacology.

[B28] Todorova A, Samuel M, Brown RG, Chaudhuri KR (2015). Infusion therapies and development of impulse control disorders in advanced parkinson disease: clinical experience after 3 years' follow-up. Clin Neuropharmacol.

[B29] O'Sullivan SS, Evans AH, Lees AJ (2009). Dopamine dysregulation syndrome: an overview of its epidemiology, mechanisms and management. CNS Drugs.

[B30] Hughes AJ, Daniel SE, Kilford L, Less AJ (1992). Accuracy of clinical diagnosis of idiopathic Parkinson's disease: a clinico-pathological study of 100 cases. J Neurol Neurosurg Psychiatry.

